# Clinicopathological Features and Postoperative Survival Analysis of Gastric Carcinoma with Neuroendocrine Differentiation

**DOI:** 10.1155/2022/4440098

**Published:** 2022-08-17

**Authors:** Jin Liu, Xiaohua Pan, Yan Sun, Tingting Dong, Xiao Hu, Huijuan Zhong, Jianwei Lu

**Affiliations:** ^1^The Department of Oncology, The Affiliated Suqian First People's Hospital of Nanjing Medical University and Suqian First Hospital, Suqian 223800, China; ^2^The Department of Oncology, The Affiliated Cancer Hospital of Nanjing Medical University and Jiangsu Cancer Hospital and Jiangsu Institute of Cancer Research, Nanjing 210000, China

## Abstract

**Objectives:**

This study aims at investigating the differences of clinicopathological features and postoperative prognosis in three different types of neuroendocrine differentiation-related gastric cancers.

**Methods:**

From January 1, 2015 to September 30, 2016, 47 patients diagnosed with neuroendocrine differentiation-related gastric cancers were collected from 1095 patients with gastric cancer who underwent surgical treatment in the Department of Gastrointestinal Surgery, Jiangsu Cancer Hospital. Patients were followed up regularly, and the last follow-up time was October 25, 2021. A total of 38 cases met the inclusion criteria and completed follow-up. The clinicopathological characters and immunohistochemical results of these three special pathological types of gastric cancer (adenocarcinoma with neuroendocrine differentiation, mixed adenoneuroendocrine carcinoma, and neuroendocrine carcinoma of the stomach) patients were compared. Tissues from these patients were tested with immunohistochemical markers synaptophysin (Syn), chromogranin A (CgA), and Ki-67. The Kaplan–Meier method and log-rank test were used to analyze the effect of different histological types of gastric cancer on overall survival (OS). The differences in positive rates of chromogranin A (CgA) and Ki-67 were analyzed by univariate Cox regression analysis as independent risk factors that may affect the survival of gastric cancer patients.

**Results:**

Ki-67 and N staging were significantly correlated with OS in gastric cancer patients and were independent prognostic factors affecting the survival of gastric cancer patients. There was no statistical difference in OS between the two histopathological types (adenocarcinoma with neuroendocrine differentiation and mixed adenoneuroendocrine carcinoma) of gastric cancer patients. There were no significant differences in the positive rates of immunohistochemical markers Syn, CgA, and Ki-67 in gastric cancer patients with different histological types.

**Conclusion:**

The combined detection of Syn and CgA is of great value for the diagnosis of neuroendocrine differentiation-related gastric cancers, Ki-67 is of significance for the prognosis prediction of neuroendocrine differentiation-related gastric cancers, regional lymph node metastasis has a great impact on tumor prognosis, and the N staging determines the necessity of postoperative adjuvant chemotherapy for patients with neuroendocrine differentiation-related gastric cancer.

## 1. Introduction

Adenocarcinoma is the most common pathological types of gastric cancers, and other types of gastric cancers, such as adenocarcinoma with neuroendocrine differentiation, mixed adenoneuroendocrine carcinoma, and neuroendocrine carcinoma, are relatively rare [1]. Neuroendocrine cells are scattered among gastric cancer cells in the form of single or cell nests. If the neuroendocrine cells make up less than 30% of the whole cancer tissue, it is classified as gastric carcinoma with neuroendocrine cell differentiation (GCNED) [2, 3]. Cancerous tissue consists solely of neuroendocrine cells, called gastric neuroendocrine carcinoma (GNET) [4]. If the proportion of each cell types exceeds 30%, it will be classified as mixed adenoneuroendocrine (MANEC; WHO 2017 version was renamed mixed neuroendocrine-nonneuroendocrine neoplasms, MiNENs) [4]. These three special types of gastric carcinoma are difficult to distinguish by preoperative imaging examination and gastroscope. Thanks to the development and wide application of pathological immunohistochemistry, these neuroendocrine cell-related gastric cancers have been diagnosed, differentiated, and compared with common adenocarcinomas. The incidence of these three special types of gastric cancers is extremely low, and the prognosis is poor [5]. Some studies suggest that neuroendocrine differentiation plays a role in promoting gastric cancer progression, yet there is no clear understanding on the impact of neuroendocrine differentiation on the prognosis of the cancer and consensus on its treatment. In this study, the clinicopathological data of 47 patients with gastric cancer with special pathological types (20 cases of gastric adenocarcinoma with neuroendocrine differentiation, 11 cases of MANEC of the stomach, and 16 cases of GNET) were summarized, and the survival rates of these three groups were compared and the related factors affecting the prognosis of these three special gastric cancer patients were analyzed.

## 2. Material and Method

### 2.1. Data Collection

From January 01, 2015 to December 31, 2016, a total of 1095 patients who underwent radical gastrectomy for gastric cancer + D2 lymph node dissection and pathologically diagnosed gastric cancer in the Gastrointestinal Department of Jiangsu Cancer Hospital were reviewed and a total of 47 gastric cancer patients were collected from 1095 patients. The retrospective analysis method was used to summarize the clinicopathological characteristics of three groups of patients with different pathological types of gastric cancers, and the related factors affecting the prognosis were analyzed. Case inclusion criteria were as follows: (1) patients received radical gastrectomy + D2 lymph node dissection; (2) pathologically confirmed three special pathological types of endocrine-related gastric cancer (GCNED, and MANEC of the stomach and neuroendocrine carcinoma). The specimens of these patients were evaluated by immunohistochemical examination of neuroendocrine markers synaptophysin (Syn) and chromoprotein A (CgA); (3) aged 18–85 years; (4) postoperative pathological stage was II or III (according to AJCC 8th edition gastric cancer staging criteria); (5) all organs function normally, without serious underlying diseases; and (6) according to postoperative pathological staging, postoperative adjuvant chemotherapy is performed according to staging standards. Patients were excluded if they have other malignant tumors and serious underlying diseases made it impossible to complete the follow-up. All patients were followed up for 5 years. A total of 38 patients met the inclusion criteria and completed follow-up. Among them, 20 cases were pathologically diagnosed with gastric adenocarcinoma with neuroendocrine differentiation, 11 cases gastric mixed adeno-endocrine carcinoma, and 16 cases gastric neuroendocrine carcinoma.

### 2.2. Observation Indicator

#### 2.2.1. Clinical Data

Clinical and pathological data of patients including gender, age, smoking history, drinking history, primary tumor site, ECOG score, surgical method, preoperative tumor markers CEA and CA199, histological type, TNM stage, tumor cell differentiation degree, Syn, CgA, and Ki-67 staining outcomes were collected.

#### 2.2.2. Immunohistochemical Staining

The neuroendocrine specific markers Syn and CgA were detected immunohistochemically based on the morphological characteristics of the specimens after HE staining. If the Syn and/or CgA staining is positive, the cells were considered neuroendocrine cells. When neuroendocrine cells are less than 30% in cancer cells, it is classified as gastric cancer with neuroendocrine differentiation. Ki-67 represents nuclear proliferation index, ki-67 > 20% is set as positive, and less than or equal to 20% is set as negative.

#### 2.2.3. Postoperative Treatment and Follow-Up

Patients with stage II and above (according to the 8th edition of AJCC Gastric Cancer Staging Criteria) received postoperative adjuvant chemotherapy. Among them, the gastric neuroendocrine carcinoma group received etoposide + platinum regimen. The other two groups received fluorouracil + platinum drug regimen. All patients were followed up through the medical record system and telephone, and the follow-up ended on October 25, 2021. The follow-up content included the time of death and recurrence. Overall survival (OS) was defined as the time from the patient's pathological diagnosis to death or the end of follow-up.

### 2.3. Statistical Analysis

Statistical analysis was performed using SPSS version 18.0 software. Measurement data are expressed as *x* ± *s*, and enumeration data are expressed as rate (%). The comparison of enumeration data is by the chi-square test. The survival analysis was by the Kaplan–Meier method and log-rank test, and prognostic variables that may affect the survival time of gastric cancer patients are analyzed by univariate Cox regression analysis. *P* < 0.05 was considered to be statistically significant.

## 3. Result

### 3.1. Clinicopathological Features of the Patients

A total of 38 patients with gastric cancer were included in this study, including 33 males and 5 females from 31 to 81 years old. The mean age was 62.61 years (standard deviation 9.72), and the median age was 62.5 years; 57.89% were aged ≤65 years; 28.95% had a history of smoking, and 18.42% had a history of alcohol drinking. All subjects were assessed according to the grading criteria by Eastern Cooperative Oncology Group (ECOG) and scored 0. The mean preoperative CEA was 3.6 (standard deviation 3.7), and the mean preoperative CA199 was 232.1 (mean 1354.5). Patients with T1∼T2 and T3∼T4 staging accounted for 21.05% and 78.95%, respectively; in N staging, N0 and N1∼N3 staging accounted for 50.00% each; M staging of all patients was M0; for TNM staging, stage I-II and stage III patients accounted for 65.79% and 34.21%, respectively. Proximal gastrectomy patients accounted for 55.26%, and the rest were distal gastrectomy or total gastrectomy. In 50.00% of the patients, the primary lesions were located in the esophagus and cardia, and in 50.00% of the patients, the primary lesions were in the gastric body or gastric antrum. Adenocarcinoma with neuroendocrine differentiation, adenocarcinoma, and MANEC of the stomach and neuroendocrine carcinoma accounted for 42.11%, 21.05%, and 36.84%, respectively. Poorly differentiated gastric cancer patients accounted for 47.37%. Patients tested positive for Syn, CgA, and Ki-67 were 44.74%, 76.32%, and 52.63% of all patients, respectively. The patients with death outcome accounted for 34.21%, and the patients who survived or censored accounted for 65.79% (Table 1).

### 3.2. Immunohistochemical Analysis

**Figure 1:**
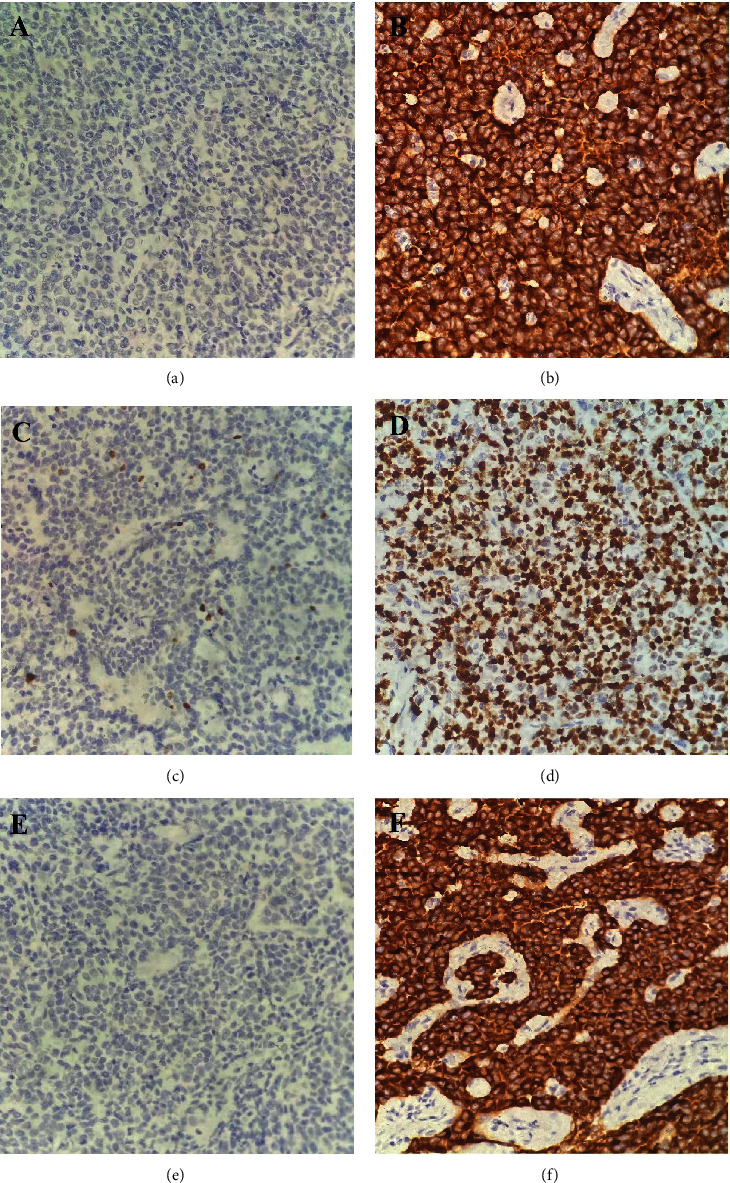
Immunohistochemical test results of gastric cancer patients. ((a) CgA negative, (b) CgA positive, (c) Ki-67 negative, (d) Ki-67 positive, (e) Syn negative, (f) Syn positive.

HE staining showed that neuroendocrine cells were distributed in single cells, sheets, or nests in gastrointestinal adenocarcinoma tissues. Neuroendocrine differentiation components were marked by the immunohistochemical SP (streptavidin-peroxidase ligation) method. The differences in the positive rates of immunohistochemical markers Syn, CgA, and Ki-67 in different histological types were analyzed by the continuity-correctedchi-square test (GCNED, and MANEC of the stomach and neuroendocrine carcinoma). The results (Table 2, Figure 1) show there was no statistically significant difference in the positive rates of Syn, CgA, and Ki-67 in gastric cancer patients (*P* > 0.05).

### 3.3. Prognostic Analysis

#### 3.3.1. Survival Analysis

**Figure 2:**
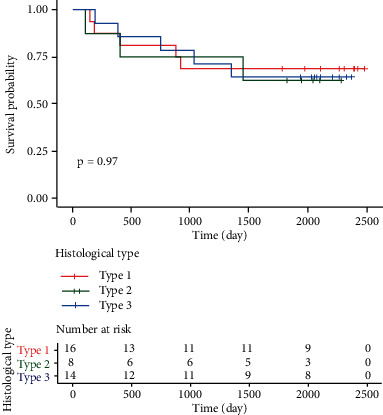
Survival curves of gastric cancer patients with different histological types. *Note*. Type 1: GCNED; type 2: MANEC of stomach; type 3: GNET.

As of the last follow-up time, 38 of the 47 patients in the whole group received complete follow-up, and the follow-up rate was 80.9%. A total of 18 patients passed away, all of them were caused by tumor recurrence and metastasis. The median OS of GCNED group was not seen, and the 1-, 3-, and 5-year survival rates were 81.25%, 68.75%, and 56.25%, respectively. The median OS was not seen in the MANEC of the stomach group, and the 1-, 3-, and 5-year survival rates were 75%, 75%, and 37.5%, respectively. The median OS of the neuroendocrine carcinoma group was not found, and the 1-, 3-, and 5-year survival rates were 85.7%, 78.6%, and 57.1%, respectively. The Kaplan–Meier method and log-rank test were used to analyze the effect of different histological types of gastric cancer on OS. The OS of patients with gastric neuroendocrine carcinoma was calculated separately due to differences in treatment regimens. The results showed (Figure 2) that there was no statistically significant difference in OS between the other two groups of gastric cancer patients with different histological types (*P*=0.97).

### 3.4. Prognostic Analysis of Three Types of Gastric Cancers

**Figure 3:**
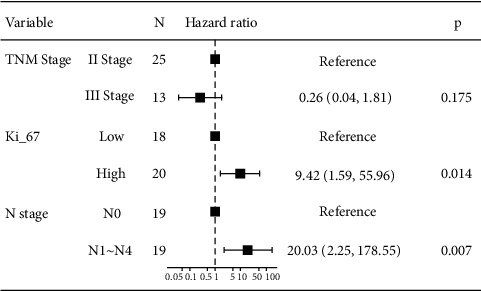
Forest plot of multivariate analysis on factors affecting the prognosis of gastric cancer (*n* = 38).

The expression of Ki-67, CgA, and Syn was analyzed by the continuity-correctedchi-square test. The results showed that there was no statistically significant difference in the positive rates of Syn, CgA, and Ki-67 in patients with three different histological types of gastric cancers (*P* > 0.05). For 16 prognostic variables that may affect the survival of gastric cancer patients, age, gender, smoking history, drinking history, T stage, N stage, TNM stage, preoperative CA199, preoperative CEA, degree of differentiation, and extent of primary tumor resection, one-way Cox regression analysis was performed on the site of primary tumor and histological type, and the results showed that in the case of a small sample size, no variable had a statistically significant effect on the OS of gastric cancer patients (*P* < 0.05) (Table 3). Then, three factors with *P* < 0.2 in univariate analysis (N stage, TNM stage, and Ki-67, *P* values of Wald test were 0.06, 0.19, and 0.15, respectively) were used in this Cox multivariate regression model analysis for further evaluation. Ki-67 and N stages were significantly correlated with OS in gastric cancer patients and were independent prognostic factors affecting the survival of gastric cancer patients (*P* < 0.05, Figure 3).

**Figure 4:**
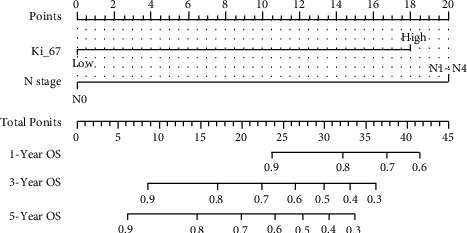
Nomogram prediction model for 1-year, 3-year, and 5-year OS in gastric cancer patients. Based on the results of multivariate analysis, combining 2 predictable indicators, we constructed a nomogram as a model for predicting 1-, 3-, and 5-year survival in gastric cancer patients.

**Figure 5:**
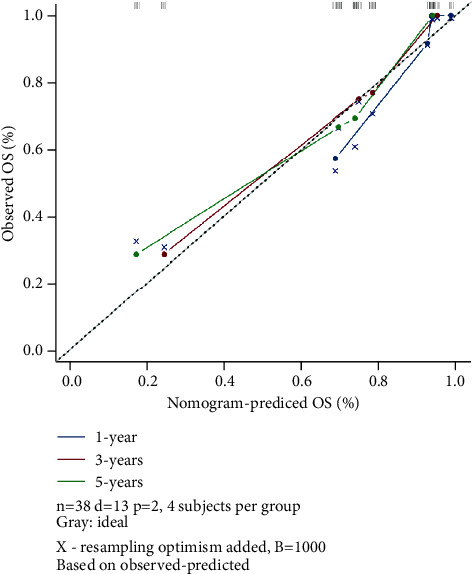
Nomogram model calibration curves for 1-, 3-, and 5-year OS in gastric cancer patients.

**Figure 6:**
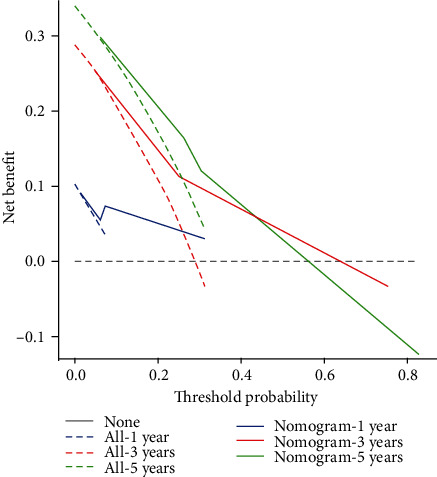
DCA for 1-year, 3-year, and 5-year OS for the nomogram model.

Based on the results of multivariate analysis, combining 2 predictable indicators, we constructed a nomogram as a model for predicting 1-, 3-, and 5-year survival in gastric cancer patients (Figure 4). The nomogram showed that N staging had the greatest impact on prognosis, and Ki-67 had a slightly lower effect on prognosis than N staging. Each level of the variable represents a different score, and the total score is obtained from the nomogram. To verify the performance of this nomogram model, we calculated the C-index and calibration curve of the model, confirming the consistency of the model. The C-index is 0.74 (95% CI, 0.61–0.86), and the calibration curve shows that our model is in good agreement with the actual observations. In this model, the *X*-axis represents the survival rate predicted by the nomogram, and the *Y*-axis represents the actual survival probability (Figures 5 and 6). Further plotting the decision curve analysis(DCA) of the 1-, 3-, and 5-year OS of the nomogram model, the *X*-axis represents the threshold probability, the *Y*-axis represents the net benefit, and the colored solid line represents the net benefit of the predicted model using the nomogram, also confirming the nomogram showed the effectiveness of predictive models (Figure 6).

Three factors with *P* < 0.2 in univariate analysis were included in this Cox multivariate regression model analysis to further explore the factors affecting the prognosis of gastric cancer patients. The results showed that Ki-67 and N stage were significantly associated with OS in gastric cancer patients and were independent prognostic factors affecting the survival of gastric cancer patients (*P* < 0.05).

The nomogram showed that N staging had the greatest impact on prognosis, and Ki-67 had a slightly lower effect on prognosis than N staging. Each level of the variable represents a different score, and the total score is obtained from the nomogram. To verify the performance of this model, we calculated the C-index and calibration curve of the model, confirming the consistency of the model. The C-index is 0.74 (95% CI, 0.61–0.86), the calibration curve shows that our model is in good agreement with the actual observations, the *X*-axis represents the survival rate predicted by the nomogram, and the *Y*-axis represents the actual survival probability (Figures 3 and 4). Further plotting the DCA of the 1-, 3-, and 5-year OS of the nomogram model, the *X*-axis represents the threshold probability, the *Y*-axis represents the net benefit, and the colored solid line represents the net benefit of the predicted model using the nomogram, also confirming the effectiveness of predictive models (Figure 5).

## 4. Discussion

At present, the main treatment for three types of neuroendocrine-related gastric cancers is surgery, combined with radiotherapy, chemotherapy, immunotherapy, and targeted therapy, but the overall prognosis is poor [6]. Although the proportion of neuroendocrine cells in the three groups of patients in this study is different, there are many similarities in clinical characteristics, such as higher percentage of male patients in each group at the time of diagnosis, which is similar to that reported by Bozkaya et al. [7]; higher percentage of patients denied smoking and drinking history; similar primary sites of the lesions; and similar lymph node metastasis [8]. Therefore, it is difficult to distinguish these three types of patients by clinical symptoms and microscopic morphology, and the only way of diagnosis is made based on the cell morphology of pathological sections and specific indicators of immunohistochemical markers.

The clinical diagnosis of GCNED and neuroendocrine carcinoma mainly relies on immunohistochemical staining [9]. In this study, the neuroendocrine markers CgA and Syn were recommended by the Chinese gastroenteropancreatic neuroendocrine tumor pathology expert group [10, 11]. In this study, we found that the positive rates of CgA in the three groups of gastric cancer patients were 68.75%, 87.5%, and 78.57%, respectively. The positive rates of Syn in the three groups of gastric cancer patients were 37.5%, 50%, and 50%, respectively. The positive expression rate of Syn was significantly higher than that of CgA, and the sensitivity was higher. Although the positive rates of the two markers were high, they did not reach 100%, so we believe that the clinical application of the combined detection of the two markers is helpful for the diagnosis of gastric cancer patients with neuroendocrine differentiation.

At present, experts and scholars in China and abroad are still debating on the influence of neuroendocrine differentiation components on the prognosis of tumor patients. Some scholars have found that the postoperative survival time of gastric cancer patients with neuroendocrine differentiation (NED) is shorter than that of patients without NED, and the difference is statistically significant. This might imply that neuroendocrine differentiation plays a role in promoting gastric cancer progression [12, 13]. Some scholars believe that the survival time of tumor patients with high expression of neuroendocrine markers CgA and Syn is significantly lower than that of patients with low expression. This may be related to the fact that neuroendocrine cell components in cancer tissue secrete various active substances through various secretory pathways, stimulate the growth of surrounding tumor cells, and enhance the proliferation and anti-apoptotic ability of cancer cells [14]. In this study, patients with three types gastric cancers that had different neuroendocrine differentiation degrees were followed up. Considering the differences in treatment regimens, The type of GNET is analyzed separately and it was found that there was no significant difference in the 1-, 3-, and 5-year survival rates of the other two groups of patients. Univariate analysis showed that the expression of CgA and Syn was not related to OS, suggesting that the prognosis of gastric cancer patients with neuroendocrine differentiation was not affected by the expression of neuroendocrine markers CgA and Syn.

In recent years, the Ki-67 proliferation index has been widely used to evaluate the proliferation ability of tumor cells, but its prognostic relevance in patients with neuroendocrine differentiation-related gastric cancer is still unclear. In breast cancer, the Ki-67 proliferation index is an independent risk factor for OS [15, 16]. Similarly, a retrospective study showed that high expression of Ki-67 in early gastric cancer was associated with poor prognosis [17, 18]. However, the role of Ki-67 in predicting prognosis is still controversial, and some scholars believe that the Ki-67 proliferation index cannot predict the clinical outcome of gastric cancer patients [19]. The results of this study found that Ki-67 was significantly associated with OS in patients with neuroendocrine differentiation-related gastric cancer and was an independent indicator of prognosis. Therefore, Ki-67 has guiding significance for the prognosis of neuroendocrine differentiation-related gastric cancer.

Postoperative adjuvant therapy for patients with TNM stage II has been controversial. From the results of this study, N stage is significantly correlated with OS in gastric cancer patients, indicating that gastric cancer patients with TNM stage II have poor prognosis if they have more regional lymph node metastasis, and N stage is an independent indicator that affects prognosis. This provides a strong basis for predicting whether postoperative adjuvant therapy is necessary based on lymph node metastasis for such neuroendocrine differentiation-related patients with TNM stage II.

In recent years, the incidence of GNET has been on the rise [20]. For metastatic and poorly differentiated GNET, a combination regimen similar to small cell lung cancer is usually used in the first-line regimen and postoperative adjuvant therapy [21]. Relevant literature reports show that the median survival time of GNET is 8–33 months, the average survival time is 14.9–40.1 months, and the 5-year survival time rate is 30%–60% [22]. In this study, we were not able to compare GNET with the other two gastric cancers in survival time and OS. In follow-up studies, we look forward to collect more cases to further explore the differences in survival and OS between GNET and the other two types of gastric cancer. In conclusion, patients with neuroendocrine-related gastric cancer are similar in age of onset, gender, and primary site of the lesion; lymph node metastasis is atypical. Many patients have lost the opportunity for surgery at the time of diagnosis, and the overall prognosis is poor. Lymph node metastasis is an independent factor for prognosis. Thus, early diagnosis, early treatment, and postoperative adjuvant chemotherapy are particularly important. The detection and diagnosis of neuroendocrine differentiation components mainly rely on immunohistochemical staining. The combined detection of CgA and Syn can improve the diagnostic rate of the disease.

## Figures and Tables

**Table 1:** Patient clinical characteristics.

	Clinical characteristics	*N* (%)
Age	≤65	22 (57.89%)
	>65	16 (42.11%)

Gender	Male	33 (86.84%)
	Female	5 (13.16%)

Smoking history	Nonsmoker	27 (71.05%)
	Smoker	11 (28.95%)

Alcohol drinking	No	31 (81.58%)
	Yes	7 (18.42%)

ECOG score	0	38 (100.00%)

Preoperative CEA	—	3.6 ± 3.7

Preoperative CA199	—	232.1 ± 1354.5

T Staging	T1∼T2	8 (21.05%)
	T3∼T4	30 (78.95%)

N staging	N0	19 (50.00%)
	N1∼N3	19 (50.00%)

M Staging	M0	38 (100.00%)

TNM staging	II	25 (65.79%)
	III	13 (34.21%)

Primary tumor resection range	Proximal stomach	21 (55.26%)
	Distal stomach or whole stomach	17 (44.74%)

Primary lesion	Esophagus + cardia	19 (50.00%)
	Body or antrum	19 (50.00%)

Histological type	GCNED	16 (42.11%)
	MANEC of the stomach	8 (21.05%)
	GNET	14 (36.84%)

Differentiation	Low	18 (47.37%)
	Medium to advanced	20 (52.63%)

Syn	Negative	21 (55.26%)
	Positive	17 (44.74%)

CgA	Negative	9 (23.68%)
	Positive	29 (76.32%)

Ki-67	Negative	18 (47.37%)
	Positive	20 (52.63%)

Outcome	Survived/truncated	25 (65.79%)
	Death	13 (34.21%)

*Note. N*-sample size; ECOG: Eastern Cooperative Oncology Group; CEA: carcinoembryonic antigen; CA199: carbohydrate antigen 199; Syn: synaptophysin; CgA: chromogranin A; Ki-67: proliferating cell Ki-67 nuclear antigens, Ki-67 > 20% is set as positive, and less than or equal to 20% is set as negative.

**Table 2:** Immunohistochemical staining in patients with different histological types of gastric cancers.

Immunohistochemical Markers	Histological types			*χ* ^2^	*P*
	GCNED (*n* = 16)	MANEC of the stomach (*n* = 8)	Neuroendocrine carcinoma (*n* = 14)		
Syn					
Negative	10	4	7	0.59	0.75
Positive	6	4	7		
CgA					
Negative	5	1	3	1.10	0.58
Positive	11	7	11		
Ki-67					
Negative	7	4	7	0.15	0.93
Positive	9	4	7		

*Note.* Ki-67 > 20% is set as positive, and less than or equal to 20% is set as negative.

**Table 3:** Univariate analysis on factors affecting the prognosis of gastric cancer (*n* = 38).

Factor	Variable	*N*	*z*	HR (95% CI)	*Pvalue*
Age		38	0.98	1.03 (0.97–1.10)	0.33

Gender	Male	33			
	Female	5	1.17	2.15 (0.59–7.84)	0.24

Smoking history	Nonsmoker	27			
	Smoker	11	0.10	1.06 (0.33–3.46)	0.92

Alcohol drinking	No	31			
	Yes	7	0.70	1.59 (0.44–5.81)	0.48

T Staging		8			
	—	30	−0.84	0.60 (0.19–1.96)	0.40

N staging	N0	19			
	N1∼N3	19	1.85	3.05 (0.94–9.93)	0.06

TNM staging	II	25			
	III	13	1.31	2.08(0.69–6.22)	0.19

Syn	Negative	21			
	Positive	17	0.26	1.16 (0.39–3.45)	0.79

CgA	Negative	9			
	Positive	29	−0.86	0.60 (0.18–1.94)	0.39

Ki-67	Low	18			
	High	20	1.44	2.38 (0.73–7.73)	0.15

Preoperative CA199	—	38	0.60	1.00 (1.00–1.00)	0.55
Preoperative CEA	—	38	−0.19	0.98 (0.84–1.15)	0.85

Differentiation	Low	18			
	Medium-advanced	20	−0.13	0.93 (0.31–2.78)	0.90

Primary tumor resection range	Proximal stomach	21			
	Distal stomach or whole stomach	17	−1.24	0.47 (0.15–1.54)	0.22

Primary lesion	Esophagus + cardia	19			
	Body or antrum	19	0.18	1.11 (0.37–3.29)	0.86

Histological type	GCNED	16			
	MANEC of the stomach	8	0.25	1.20 (0.29–5.03)	0.80
	GNET	14	0.15	1.10 (0.32–3.81)	0.88

*Note. N*: sample size; *z*: Wald statistic; HR: hazard ratio; 95% CI: 95% confidence interval; Syn: synaptophysin; CgA: chromoprotein A; Ki-67: proliferating cells, nuclear antigen; CA199: carbohydrate antigen 199; CEA: carcinoembryonic antigen.

## Data Availability

Emails could be sent to the following address to obtain the shared date (jliu0623@stu.suda.edu.cn).
